# On the Sustainability of Guideline Implementation

**DOI:** 10.1097/WOX.0b013e3181fdfc7a

**Published:** 2010-11-15

**Authors:** Juliane Köberlein, Julia Vent, Ralph Mösges

**Affiliations:** 1 Institute of Medical Statistics, Informatics and Epidemiology, Faculty of Medicine, University of Cologne, Cologne, Germany; 2 Department of Otorhinolaryngology, Head and Neck Surgery, University of Cologne Medical Center, Cologne, Germany

**Keywords:** allergic rhinitis, treatment, guidelines, compliance, ARIA, ENT specialists

## Abstract

**Background:**

Allergic rhinitis (AR) is a disorder associated with a high financial burden and is considered an important risk factor for the development of asthma. The ARIA guideline (Allergic Rhinitis and its Impact on Asthma) addresses this problem and provides recommendations for treating allergic rhinitis. The objective of the present analysis was to estimate the compliance with guidelines among ear, nose and throat (ENT) specialists and general practitioners.

**Methods:**

The data of 121,593 patients collected during 9 prospective observational studies carried out from 1998 to 2005 were examined using individual patient data meta-analysis method.

**Results:**

Only 14.8% of patients with allergic rhinitis were treated according to the recommendations. Of the others, 73.8% received insufficient treatment. In addition, 36.1% of the patients who were treated by ENT specialists received therapy according to guidelines, whereas only 16% of the general practitioners heeded the recommendations. Patients suffering from rhinitis and asthma were treated by ENT specialists according to the ARIA guideline in 50% of cases. It could be observed that the rate of guideline compliance was highest in the year of publication.

**Conclusion:**

The results are evidence of the successful implementation process of the ARIA guidelines. However, they have not yet found their way into the daily routine of general practitioners.

## 

Allergic rhinitis (AR) is a disease associated with a high economic burden and is also considered an important risk factor for the development of asthma [1,2]. Guidelines should give treatment recommendations to allergists, ENT specialists and general practitioners to minimize the burden of disease, the impairment of quality of life, and to limit the development of concomitant diseases [3]. The 'Allergic Rhinitis and its Impact on Asthma' (ARIA) guideline,[4] which was introduced in 2001, for example, makes treatment recommendations for allergic rhinitis. It gives treatment advices for allergic rhinitis based on the concept of "one airway, one disease."

The objective of the present analysis was to estimate the compliance with and the acceptability of these guidelines[5,6] among ENT specialists[7] and general practitioners when treating patients with allergic rhinitis. For this purpose, data from prospective postmarketing observational studies were evaluated to obtain an overview of the general practice setting.

## Materials and methods

### Identification of Relevant Noninterventional Studies

To examine physician compliance to the treatment protocol of the guidelines in daily practice, individual patient data from observational studies carried out in Germany were identified to be used in this analysis. We searched for published or unpublished patient data from noninterventional studies in which patients were treated with an antihistamine, as monotherapy or in combination with other medication, because the use of oral antihistamines is considered a corner-stone of an antiallergic therapy and is generally a first-line treatment choice. To avoid selection bias and to permit extrapolation and interpretation of the pooled study results, the relevance of trials was evaluated by defining the after inclusion criteria:

• Availability of individual patient data

• Inclusion of patients with a proven diagnosis

• Clear statement of the therapeutic regimen (eg, dosage, frequency, type of application)

• Use of new generation oral antihistamine (desloratadine, ebastine, fexofenadine, levocetirizine) as monotherapy or in combination with intraocular chromones or H_1_-blocker, systemic or intranasal corticosteroids, intranasal H_1_-blocker or decongestants

• A duration of treatment of at least 2 weeks

A total of ten noninterventional studies (all still unpublished at the beginning of this evaluation in 2007) were identified, of which 9 met the inclusion criteria and were used for further analysis.

### Physicians

To estimate ENT specialists' and general practitioners' acceptance of guidelines and compliance with them, the treatment practices of about 22,000 study sites were evaluated. There were 1,000 ENT specialists and 3,506 general practitioners included in the comparison between the 2 groups, in terms of insufficiency, excess, or incorrectness of prescribed treatment.

### Patients

The data of 121,593 patients (aged 12-103 years) with a proven diagnosis of seasonal allergic rhinitis (SAR) were analyzed. Patients were also included if they suffered from concomitant asthma. Because of the observational character of the trials, no strict inclusion and exclusion criteria could be applied. The patient selection was based on the recommendations in the summary of the product characteristics (SmPC).

### Outcome Measures

To assess physician guideline compliance, the frequency of insufficient (undertreatment), excessive and incorrect treatment was measured. A treatment regimen was assessed as insufficient, if a patient didn't receive the required add-on therapy. A therapy was evaluated as excessive, if a patient received an add-on therapy or further medication while this was not necessary. Did the patient sustain damage while receiving an insufficient or excessive treatment, therapy was assessed as "incorrect treatment."

The similar methods of documentation of medications prescribed and symptom severity in each study made it possible for us to develop a common algorithm with which we could assess the extent of physician adherence to guidelines (Table 1). This algorithm illustrates the smallest common denominator of all available guideline [1-6].

**Table 1:** Assessment of guideline conformity

	Nasal symptoms	
	
	Mild	Moderate Severe
Nasal obstruction Mild	Antihistamines	Antihistamines *and *intra-nasal corticosteroid
Moderate severe	Antihistamines *and *intra-nasal decongestant or intra-nasal corticosteroid	Antihistamines *and *intra-nasal decongestant and/or intra-nasal corticosteroid

Furthermore, the difference between compliance of ENT specialists and general practitioners with guidelines was analyzed. Because the postmarketing studies were conducted in different years (first 1998, last 2005), it was also possible to examine the change in the physicians' compliance with national and international guidelines over time.

### Data Analysis

To avoid any bias upon data consolidation, only variables that were assessed in all 9 studies were taken for analysis. The protocol predefined the processing of the different variable groups both during and after the synthesis process. After data synthesis, the data sets were examined for plausibility and errors. Whenever it was not possible to rectify the variables of a data set, missing values were assumed instead. After controlling for plausibility, patient data sets with less than 50% of all data necessary for calculation of analysis parameters were identified, to be excluded from the final analysis file. The frequency of insufficient, excessive and incorrect treatment was determined using the algorithm described above.

Each of the 9 studies was first analyzed individually. Then, subsequent analysis of the total data pool was performed to obtain descriptive statistics. Data were checked for homogeneity of demographic characteristics among the different studies, and ordinal parameters were examined by means of contingency tables. Both the Kruskal-Wallis test and the Mann-Whitney *U *test were used to examine differences between studies and to explain heterogeneous.

## Results

The data of 121,563 patients was analyzed. Overall, 76,981 patients (mean age 37.70 years ± 14.62) were included in the final analysis. Fifty-five of the patients were female and 45% male. Furthermore, data of 2,259 patients with a concomitant asthma were enrolled; 44,582 patients were excluded because of incomplete treatment documentation.

**Figure 1:**
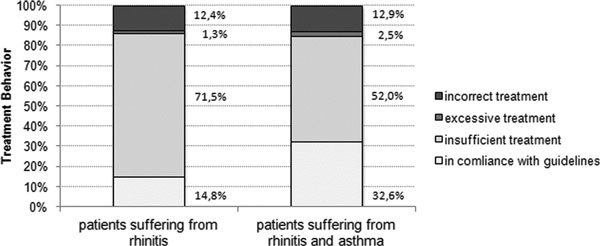
**Guideline compliance in the treatment of patients suffering from rhinitis and patients with concomitant asthma**.

**Figure 2:**
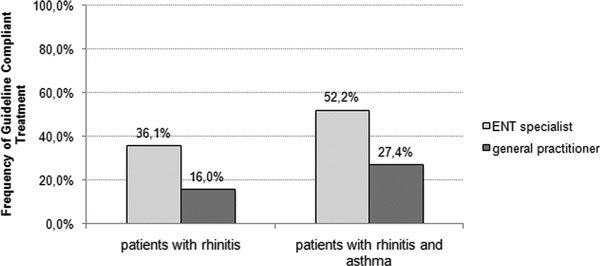
**Frequency of guideline compliant treatment differentiating between the prescribing physicians (general practitioner or ENT specialist)**.

The overall guideline analysis showed that only 14.8% of patients with allergic rhinitis were treated according to recommendations, 71.5% percent were treated insufficiently and 12.4% received incorrect therapy. Patients with concomitant asthma were treated in compliance with the guidelines in only 32.6% of cases. Fifty-two percent received insufficient and 12.9% incorrect treatment (Figure 1). Comparison between ENT specialists and general practitioners demonstrated that 36.1% of patients treated by ENT specialists received therapy according to these international guidelines. In contrast, only 16% of the general practitioners followed the guideline recommendations (Figure 2).

Next, the guideline compliance of ENT specialists when treating allergic patients with concomitant asthma in studies conducted after 2001 was analyzed, because the ARIA guideline, published 2001 especially deals with patients suffering from asthma concomitantly. The results demonstrate that more than 50% of patients with rhinitis and concomitant asthma were treated according to the guidelines.

**Figure 3:**
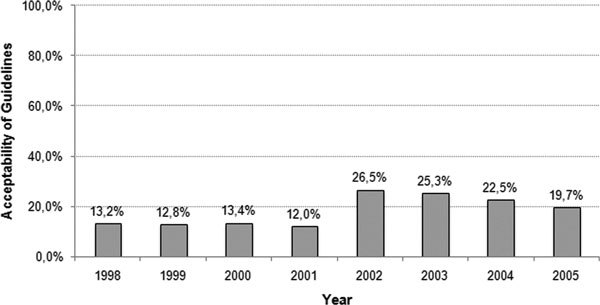
**Timeline of guideline acceptability**.

The authors also examined the change in the physician's treatment choices in general, regardless of their specialty. It was observed that the rate of guideline compliance and acceptance in 2002 was higher than that in previous years. In this year, after the publication of the ARIA document, 26.5% of all physicians complied with the guideline (Figure 3). The authors presume that this rise was attributed to the awareness campaign promoted during the implementation process of the ARIA guideline.

## Discussion

The authors carried out an individual patient data (IPD) meta-analysis of open, prospective observational studies in patients suffering from allergic rhinoconjunctivitis with and without asthma, to analyze physician's adherence to current guidelines. The analyses demonstrated that less than a quarter of patients received the treatments recommended in the guideline. Furthermore, a comparison between ENT specialist and general practice demonstrated that only 16% of the general practitioners followed the guideline recommendations (vs. 36.1% of ENTs).

Today, most guidelines for the treatment of allergic rhinitis primarily give treatment recommendations to physicians from an ENT specialist's point of view. The present investigation shows that the ARIA statement[4] has not yet found its way into the daily routine of general practitioners in Germany and elsewhere in Europe. ENT specialists apply the recommendations more readily in practice.

This finding thus supports the goals set by the "International Primary Care Respiratory Group" for implementing special guidelines for general practitioners in the primary care setting [6].

Another fact to be pointed out is the good compliance with guidelines in the therapy for allergic patients with concomitant asthma. More than 50% of allergic patients suffering from concomitant asthma (and thus a more severe disease) received treatment according to the guidelines. Further, studies conducted after 2001 were examined. Based on these results, we therefore presume that the good compliance with guidelines is related to the treatment recommendations of the ARIA guidelines that take into account the concept "one airway, one disease."[4] Thus, adherence to the ARIA guidelines for the treatment of allergic upper airway disease is poor. In their best year of implantation only 26.5% of patients were treated accordingly. Regarding the timeline of guideline acceptability, the results are evidence of the well-structured and successful implementation process of the ARIA guidelines. In the year 2002, the acceptability of guidelines among physicians doubled from 12% (2000) to 26.5% (2001/2002). There is a high necessity of disseminating guidelines over a longer period of time. Furthermore, it is essential to shorten the interval between guideline updates to strengthen confidence in such recommendations.

Despite the possibility to study physician's treatment behavior under real-world conditions the difficulties of non-interventional studies have to be discussed. This type of study can usually be better generalized, but data may be biased in other ways. While interpreting the presented results one has to take into consideration that the primary objectives of the included noninterventional studies were originally established to evaluate the effectiveness of antihistamines under real-world conditions. Furthermore, these observational studies were conducted by the companies. Therewith, no specific algorithm for study site recruitment was used to ensure a representative population sample of practitioners and patients for a guideline implementation discussion. Hence, the data are inappropriate to demonstrate in detail and without any bias, which implementation and application problems exist. In addition to that, the use of existing study data creates a high patient exclusion rate of nearly 40%.

Nevertheless, the analyzed data do however allow indication of potential system or organizational caused deficits. A health services research study should rather be conducted to provide valid information about the real-world implementation and the suitable application process and to give guidance to further optimization of the guideline.

Therefore, it should be the aim of each guideline expert panel to conduct an extensive study program, comprising a randomized controlled trial and a health services research study, to detect as accurately as possible factors that may influence effectiveness and efficiency of guideline recommendations.

## Note

Meetings at which parts of the data were presented: XX. World Allergy Congress 2007, Bangkok: 2-6 DEC. 2007; AAAAI-Kongress 2007, San Diego: 22-27 FEB. 2007, CA.
